# A Simple and Efficient Method for Preparing Cell Slides and Staining without Using Cytocentrifuge and Cytoclips

**DOI:** 10.1155/2015/813216

**Published:** 2015-11-17

**Authors:** Xiaotang Hu, Verronika Laguerre, Daniel Packert, Alice Nakasone, Lynn Moscinski

**Affiliations:** ^1^Department of Biology, College of Arts & Sciences, Barry University, Miami Shores, FL, USA; ^2^Biomedical Sciences Program and Clinical Biology, College of Nursing and Health Sciences, Barry University, Miami Shores, FL, USA; ^3^Division of Hematopathology and Laboratory Medicine, Department of Oncologic Sciences, University of South Florida and H. Lee Moffitt Cancer Center, Tampa, FL, USA

## Abstract

Cell staining is a necessary and useful technique for visualizing cell morphology and structure under a microscope. This technique has been used in many areas such as cytology, hematology, oncology, histology, virology, serology, microbiology, cell biology, and immunochemistry. One of the key pieces of equipment for preparing a slide for cell staining is cytology centrifuge (cytocentrifuge) such as cytospin. However, many small labs do not have this expensive equipment and its accessory, cytoclips (also expensive relatively), which makes them difficult to study cell cytology. Here we present an alternative method for preparing a slide and cell staining in the absence of a cytocentrifuge (and cytoclips). This method is based on the principle that a regular cell centrifuge can be used to concentrate cells harvested from cell culture and then deposit the concentrated cell suspension to a slide evenly by using a cell spreader, followed by cell staining. The method presented is simple, rapid, economic, and efficient. This method may also avoid a possible change in cell morphology induced by cytocentrifuge.

## 1. Introduction

In both basic research and clinical investigation/diagnosis, cell staining is a necessary and useful technique to visualize cell morphology and structure under a microscope. This technique has been used in many areas such as cytology, hematology, oncology, histology, virology, serology, microbiology, cell biology, and immunochemistry. In a basic research lab, cell morphology study, in a standard protocol, involves three steps: (1) cell culture and treatment, (2) preparation of slides, and (3) cell staining and visualization. The preparation of the slides usually uses cytoclips (or called slide clips) to hold the slide with a slide funnel (cytology funnel) together, so that cell suspension can be dropped into the funnel. Then, the slide-funnel complex is set for centrifugation in a centrifuge especially designed for slides (cytocentrifuge) [1–5]. The purpose of the centrifugation is to concentrate and deposit cells from the funnel to the slide evenly relatively. After centrifugation, the slides are immersed in several bottles that contain either stains or water for staining and washing steps, one by one, in a specific consecutive order. Since a cytocentrifuge and (metal) cytoclips are relatively expensive, these prevent many small labs or individual researchers from working in the area of cell cytology. In addition, the cytospin-induced changes in cell morphology have been reported and cytospin smears should be evaluated with caution [2]. Here we present a simple and efficient alternative method for preparing cell slides and staining in the absence of a cytocentrifuge and cytoclips. This method is based on the principle that a regular cell centrifuge (all labs dealing with cell culture have this equipment) can also be used to concentrate cells harvested from cell culture and then deposit the concentrated cell suspension to a slide evenly by using a cell spreader, after which the slides are ready for staining. This method avoids cytospin-induced possible change in cell morphology. The staining procedure we used also bypasses several steps used in a standard protocol. Our results demonstrated that this new method is simple, efficient, and economic. The slides made by this method have the same applications in cytology study as the one prepared from a cytocentrifuge but avoid possible changes in cell morphology induced by cytospin method. We have used these noncytospin slides to observe Phorbol 12-myristate 13-acetate- (PMA-) induced differentiation and Bay 11-7085-induced apoptosis in several human myeloid leukemia cells after staining with different stain reagents.

## 2. Materials and Methods

### 2.1. Preparation of Slides and Glass Spreader

Human myeloid leukemia cells lines, TF-1a, TF-1, and MV4-11, were purchased from ATCC (Rockville, MD) and maintained in RPMI 1640 (TF-1a and TF-1) or IMDM (MV4-11) supplemented with 10% FBS without (TF-1a) or with GM-CSF (5 ng/mL, TF-1, MV4-11) at 37°C in humidified air containing 5% CO_2_. All the media and sera were purchased from Life Technologies, Inc. (Gaithersburg, MD). During log-phase growth, the cells were collected and transferred to 6-well plates at a concentration of 10^5^ cells/mL. For differentiation study, PMA (Sigma Chemical Co., St. Louis, MO) was added to the cells and incubated for 48–72 h. For apoptosis assay, cells were treated with Bay 11-7085 (Calbiochem, La Jolla, CA) at different concentrations. The control cells were treated with PMA or Bay 11-7085 solvent. After the treatment for 24 and 48 hours, the cells were harvested into a 15 mL polypropylene centrifuge tube and spun down for 8 min at 600 RPM (80 g), after which the supernatant was discarded and the cells were resuspended in 0.5 mL of culture medium and 1-2 drops of the cell suspension were placed on a slide in the central area and moved around to form a thin and even film with a glass spreader. The glass spreader was made from glass transfer pipette over an alcohol burner for few seconds to minutes.

### 2.2. Cell Staining

Two types of stain methods were used in this study: Giemsa and methylene blue/eosin. For methylene blue/eosin staining, the slides were fixed by placing 2 drops of fixing solutions on the slides and air dried in a biological hood (2 min) or on bench (3–5 min). Next, two drops of methylene blue (1%) were added to the slides and left on for 2 min, after which the excess stains were removed by placing a piece of paper towel on the stain briefly (1 second). Subsequently, two drops of 1% eosin were added to the slides and incubated for 2 min at room temperature. The slide was rinsed briefly with small amounts of tap water, after which one small drop of mounting medium was added to the slide and covered with a coverslip. For Giemsa staining, 2 drops of 5% Giemsa were added to a fixed slide followed by the procedure described for methylene blue, omitting the steps for second stain. In order to test whether the slides prepared by this method are good for commercial stain kit, we also used the DIPP KWIK Differential Stain Kit (American MasterTech, Lodi, CA).

## 3. Results and Discussion

**Figure 1:**
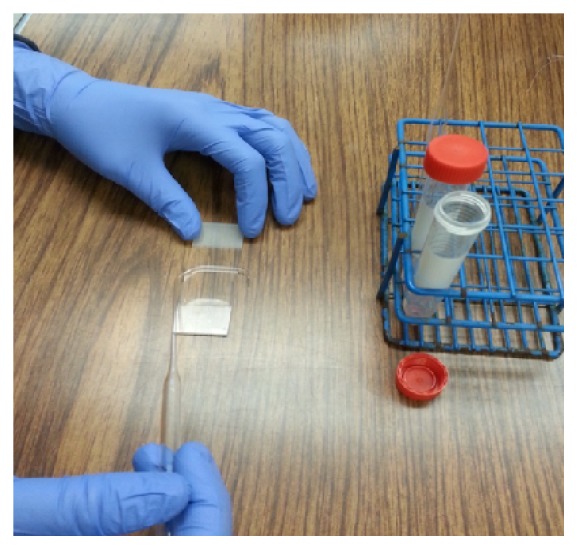
Spreading cells on a slide with a glass spreader. The glass spreader was made from a glass transfer pipette by heating it for a few seconds to minutes over an alcohol burner.

**Figure 2:**
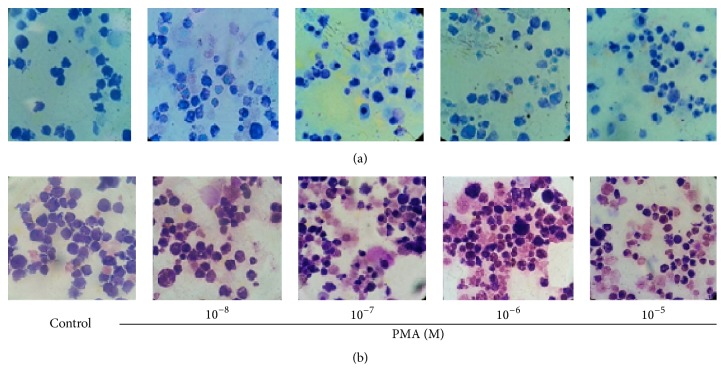
PMA induces macrophage-like differentiation. TF-1a cells were treated with PMA for 72 hours, after which the cells were collected and slides were prepared and then stained following the protocol described in Section 2. (a) Giemsa staining; (b) (×1000): methylene blue/eosin staining (×1000).

**Figure 3:**
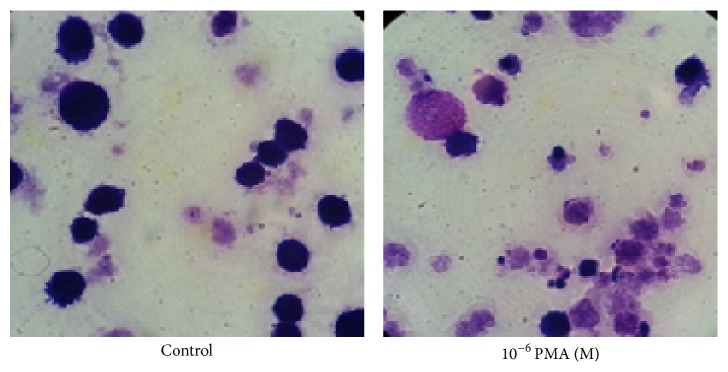
TF-1a cells stained with DIPP Quick Stains. TF-1a cells were treated with PMA for 48 hours, after which the cells were collected and slides were prepared following the method described in Section 2. The staining method followed manufacture's instruction (×1000).

**Figure 4:**
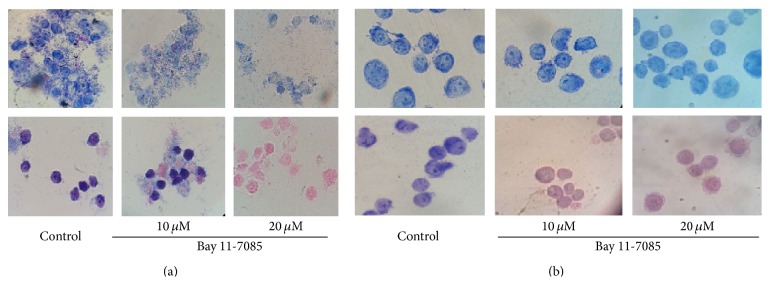
Bay 11-7085 induces apoptosis in TF-1 but not in MV4-11 cells. Log-phase TF-1 (a) and MV4-11 (b) cells were treated with Bay 11-7085 for 24 hours, after which the cells were collected and slides were prepared and stained following the protocol described in Section 2. Top panel: Giemsa staining (×1000); bottom panel: methylene blue/eosin staining (×1000).

In a basic research, the cell concentrations in culture are usually under 10^6^/mL in order to prevent contact inhibition; in particular, the cells need to be treated with growth factors, interleukins, and stimuli for few hours to days. Therefore, these cells need to be concentrated before applying them to a slide. In the absence of a cytology centrifuge, we used regular cell centrifuge to concentrate cells harvested from the culture (see Materials and Methods). Subsequently, the 1-2 drops of the concentrated cells were dropped to the central area of the slide with a plastic transfer pipette, after which a glass spreader was used to move cells around to form a thin and even layer. The glass spreader was made from a glass transfer pipette over an alcohol burner for a few seconds to 1 min.  Figure 1 shows an image of the glass spreader during the process of applying cells to a slide. In order to test whether the slides made by using this noncytocentrifuge method and the glass spreader are good enough for cytology study, three types of human myeloid leukemia cells were applied for preparation of slides and two types of stain methods were used for staining. The first cell line is TF-1a. TF-1a is a factor-independent cell line derived from the human factor-dependent erythroid leukemia TF-1 cell line but appears less mature than TF-1, because TF-1a is CD34 positive (+) and CD38 is negative (−), whereas TF-1 is CD34 + and CD38 +. TF-1a cells have big nucleus/cytoplasm ratio in the absence of differentiation. Our previous experiments have demonstrated that TF-1a cells are able to respond to PMA and can be induced to macrophage-like differentiation [6]. This cell line is a good model to study cell differentiation. As shown in Figure 2, cells treated with Giemsa (a) show good contrast. The dye stains cytoplasm, membrane, and nuclei. After addition of PMA to the cells for 72 h, the ratio of nucleus/cytoplasm is significantly reduced. The cells in (b) are stained with methylene blue and eosin. The methylene blue stain makes nuclei more visible although it stains both nuclei and cytoplasm; the eosin mainly colors the cytoplasm and cell membranes. From Figure 2, 90% of the control cells (without PMA) show a blue color, indicating the majority of the cells are nucleus, which gives a larger ratio of nucleus/cytoplasm. After treatment of the cells with PMA for 72 hours, the pink color from the eosin stain becomes dominant indicating the major part of the cells has changed to cytoplasm due to a decrease in the size of nucleus, leading to a smaller ratio of nucleus/cytoplasm. Next, we tested whether the slides prepared by this method gave equivalent effect to a commercial stain kit; the DIPP Quick Stain was used. This kit contains two dyes: methylene and eosin. As indicated in Figure 3, the DIPP KWIK smears show similar images and color changes to those slides stained with the reagents shown in Figure 2. Overall, the smear images from the slides prepared by our noncytocentrifuge method are equivalent to the slides prepared from cytocentrifuge in our previous experiments [6]. The second cell line tested is TF-1. As described above, TF-1 is more mature than TF-1a. This cell line is insensitive to PMA-induced differentiation but can be induced to apoptosis in response to Bay 11-7085, a NF-*κ*B inhibitor that has been reported to induce cell death in several cell types [7, 8].  Figure 4(a) shows the morphology of TF-1 cells in the absence or presence of Bay 11-7085 for 48 hours. The control cells (without Bay 11-7085) show normal cell morphology with complete cell membrane. The treatment of the cells with Bay 11-7085 caused significant changes in the cell morphology. At a concentration of 10 *μ*M, Bay 11-7085 caused the cells shrinkage and blebbing. About 50% of cells were completely broken down when the concentration was increased to 20 *μ*M (Figure 3). The third cell line tested is MV4-11 cells. MV4-11 is CD38+/CD34+ and is the most matured cell line as compared with TF-1a and TF-1. This cell line does not respond to PMA-induced differentiation and Bay 11-7085 induced apoptosis. There are no significant changes in the morphology between the control cells (without Bay 11-7085) and the cells treated with Bay 11-7085 for 48 hours (Figure 4(b)).

## 4. Conclusions

Taken together, our results demonstrate that this simple, economic, and time-saving method is very useful in analyzing cell cytology in the absence of a cytology centrifuge, which will benefit many small basic research labs and individual researchers. Furthermore, this noncytocentrifuge method avoids a possible cytospin-induced change in cell morphology. Although this study focuses on the cytology study with two stain reagents and one commercial stain kit, using three types of leukemia cells, we believe that the slides made by this noncytocentrifuge method can also apply to the study in immunohistochemistry and immunofluorescence areas. However, this method is not suitable for the samples with very low concentrations of cells and there is no way to grow these cells.
